# A diagnostic challenge of advanced-stage cardiac Fabry disease without left ventricular hypertrophy: a case report

**DOI:** 10.1093/ehjcr/ytag234

**Published:** 2026-03-20

**Authors:** Masataka Suzuki, Yuko Yoshigai, Hiromi Hashimura, Takeshi Aiba, Seiko Ohno, Tsuyoshi Osue, Hiroshi Eizawa, Hidekazu Tanaka

**Affiliations:** Department of Cardiology, Kobe City Nishi-Kobe Medical Center, 5-7-1, Kojidai, Nishi-ku, Kobe, Hyogo 651-2273, Japan; Department of Cardiology, Kobe City Nishi-Kobe Medical Center, 5-7-1, Kojidai, Nishi-ku, Kobe, Hyogo 651-2273, Japan; Department of Radiology, Kobe University Graduate School of Medicine, 7-5-2 Kusunoki-cho, Chuo-ku, Kobe, Hyogo 650-0017, Japan; Department of Cardiovascular Medicine, National Cerebral and Cardiovascular Center, 6-1 Kishibe-shimmachi, Suita, Osaka 564-8564, Japan; Medical Genome Center, National Cerebral and Cardiovascular Center, 6-1 Kishibe-shimmachi, Suita, Osaka 564-8564, Japan; Department of Cardiology, Kobe City Nishi-Kobe Medical Center, 5-7-1, Kojidai, Nishi-ku, Kobe, Hyogo 651-2273, Japan; Department of Cardiology, Kobe City Nishi-Kobe Medical Center, 5-7-1, Kojidai, Nishi-ku, Kobe, Hyogo 651-2273, Japan; Division of Cardiovascular Medicine, Department of Internal Medicine, Kobe University Graduate School of Medicine, 7-5-2 Kusunoki-cho, Chuo-ku, Kobe, Hyogo 650-0017, Japan

**Keywords:** Case report, Cardiac Fabry disease, Left ventricular hypertrophy, Fatty infiltration, Heart failure, Multimodal imaging

## Abstract

**Background:**

Fabry disease is a rare, X-linked lysosomal storage disorder, leading to α-galactosidase A deficiency. The primary cardiac involvement is left ventricular (LV) hypertrophy (LVH), which can progress to LV dysfunction, heart failure, and fatal arrhythmias. We present a diagnostically challenging case of advanced-stage cardiac Fabry disease with worsening LV dysfunction without LVH.

**Case summary:**

A 72-year-old woman with a family history of dilated cardiomyopathy was referred for regular follow-up. The initial electrocardiogram 14 years ago showed a shortened PQ interval minus the P-wave interval and inverted T waves; however, LVH was not observed. Three years prior, echocardiography revealed inferolateral wall thinning, and computed tomography and magnetic resonance imaging confirmed fatty infiltration and transmural late gadolinium enhancement in the thinned inferolateral wall. Two years later, she was admitted for heart failure, with LV ejection fraction decreasing to 29%. Owing to progressive LV dysfunction and family history, comprehensive genetic testing was performed, identifying a pathogenic *GLA* variant c.902G>A (p.Arg301Gln) that confirmed Fabry disease. Enzyme replacement therapy was initiated.

**Discussion:**

This case demonstrates that advanced-stage cardiac Fabry disease can progress to LV dysfunction, wall thinning, and fibrosis in the absence of prior LVH. Electrocardiogram abnormalities and multimodal imaging findings, such as wall thinning, fatty infiltration, and late gadolinium enhancement, are key indicators of cardiac Fabry disease. This case emphasizes the importance of comprehensive evaluation for early diagnosis, including electrocardiography, echocardiography, computed tomography, and magnetic resonance imaging, even without LVH.

Learning points

- Left ventricular hypertrophy (LVH) is a hallmark of cardiac Fabry disease; however, myocardial injury occurs in the early stages, even without LVH.
- Cardiac Fabry disease can progress to left ventricular dysfunction without LVH in advanced stages.
- Comprehensive evaluation, including electrocardiography, echocardiography, computed tomography, and magnetic resonance imaging, is essential for early diagnosis and treatment.

## Introduction

Fabry disease is a rare, X-linked lysosomal storage disorder caused by mutations in the *GLA* gene, leading to α-galactosidase A deficiency that impairs glycosphingolipid metabolism and causes subsequent accumulation of globotriaosylceramide (Gb3) in various organs, including the heart. Cardiac involvement is a major determinant of morbidity and mortality, typically manifesting as left ventricular hypertrophy (LVH).^1^ Myocardial injury from Gb3 accumulation begins to progress even without LVH. It is crucial to make an early diagnosis by comprehensive evaluation using electrocardiography (ECG), transthoracic echocardiography (TTE), and cardiac magnetic resonance (CMR) imaging.^2^ In the advanced stage, cardiac involvement progresses from LVH to left ventricular (LV) dysfunction, leading to heart failure and fatal arrhythmias.^3^ Advanced-stage cardiac Fabry disease presenting systolic dysfunction without LVH has never been reported. Here, we present a diagnostically challenging case of advanced-stage cardiac Fabry disease with an atypical presentation lacking LVH.

## Summary figure

**Figure:**
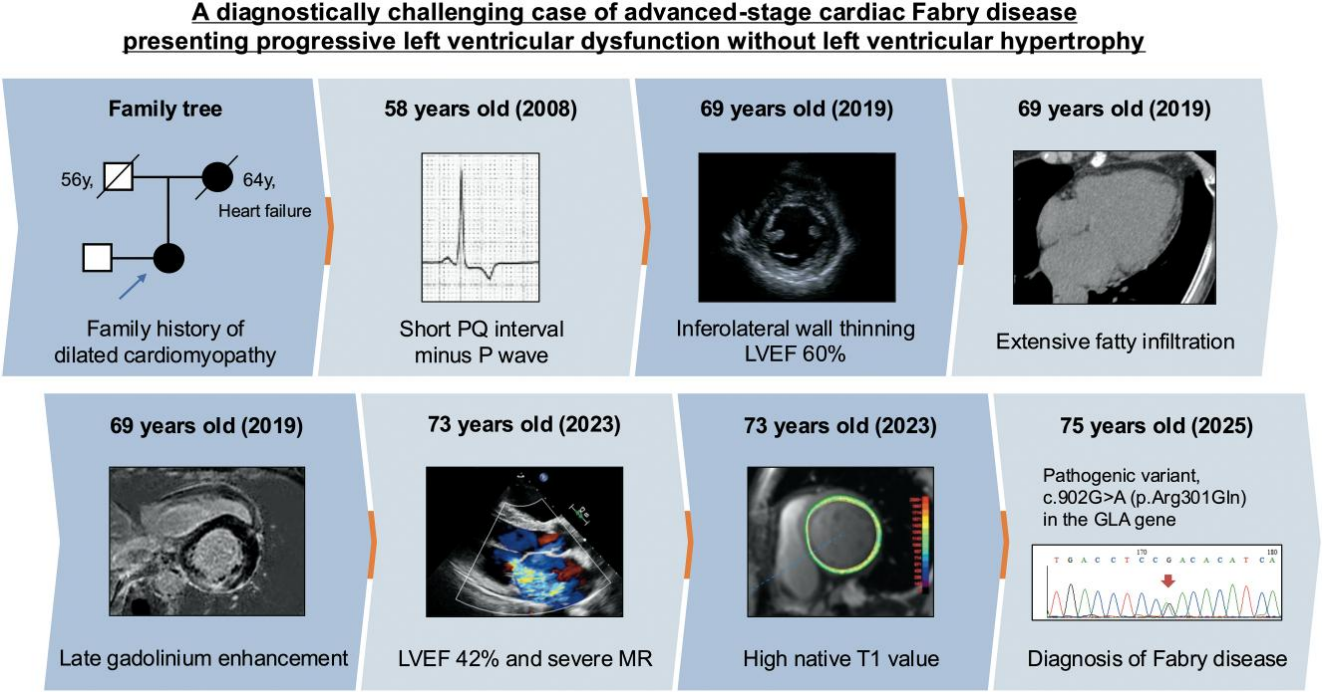


## Case presentation

**Figure 1:**
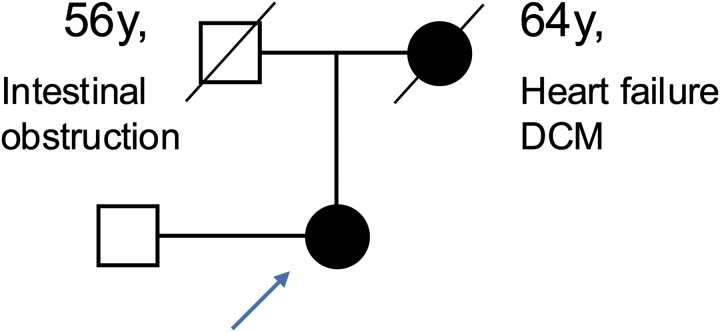
Family tree demonstrating that the patient’s mother died of heart failure owing to dilated cardiomyopathy in her 60s.

**Figure 2:**
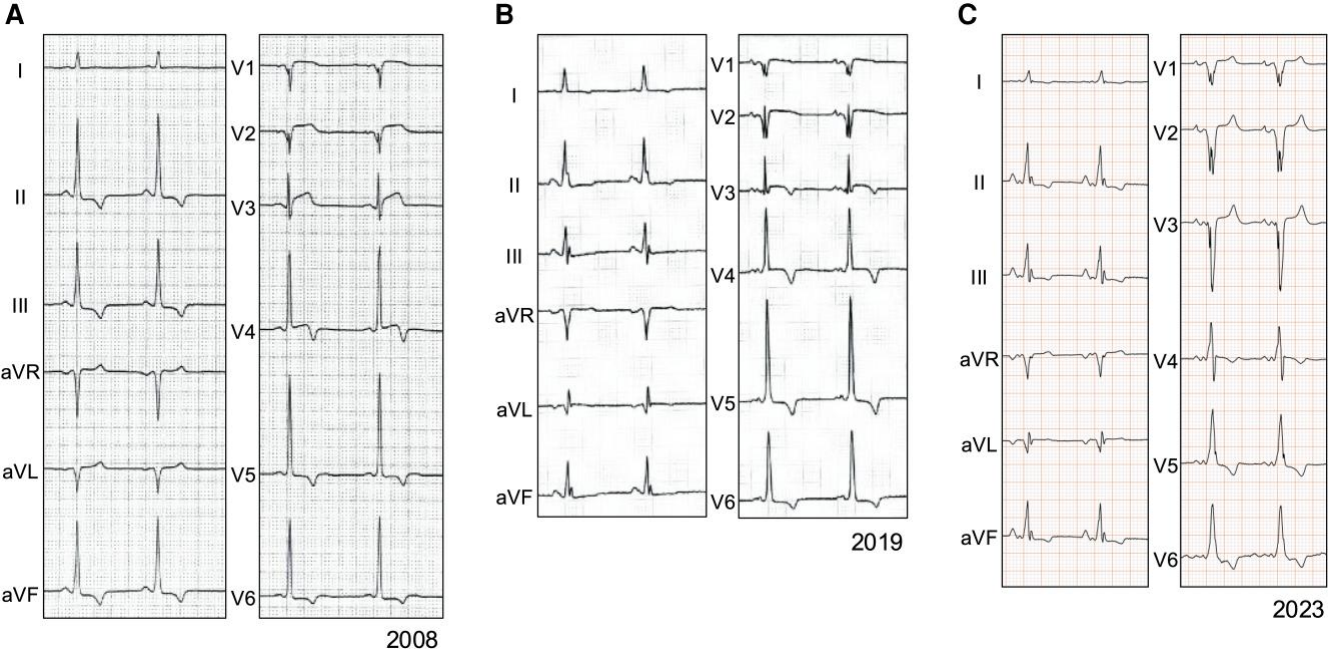
(*A*) Electrocardiogram in 2008 showing a shortened PQ interval minus P wave of 34 ms, high voltage, and inverted T waves in Leads II, III, aVF, and V4–V6. (*B*) Electrocardiogram in 2019 showing a shortened PQ interval minus P wave of 36 ms and inverted T waves in Leads V3–V6. (*C*) Electrocardiogram in 2023 showing intraventricular conduction disturbance and inverted T waves in Leads II, III, aVF, and V4–V6. Electrocardiograms were recorded with 25 mm/s and 1 cm/mV.

A 72-year-old woman with a medical history of paroxysmal atrial fibrillation was referred to our hospital for regular follow-up in 2022. The patient’s mother died of heart failure owing to dilated cardiomyopathy in her 60s; she had no children or siblings (*Figure 1*). She underwent cardiac evaluation for chest pain at a cardiology hospital in 2008. Electrocardiography revealed a shortened PQ interval minus the P wave (P_end_Q interval), high voltage, and inverted T waves in leads II, III, aVF, and V4–V6 (*Figure 2A*). Transthoracic echocardiography revealed mild mitral regurgitation (MR) without LVH or wall thinning. Coronary angiography revealed no significant stenosis or obstruction. Electrocardiography findings suggested a possibility of Wolff–Parkinson–White syndrome, but an electrophysiological study found no accessory conduction pathways. No further evaluations were performed.

**Figure 3:**
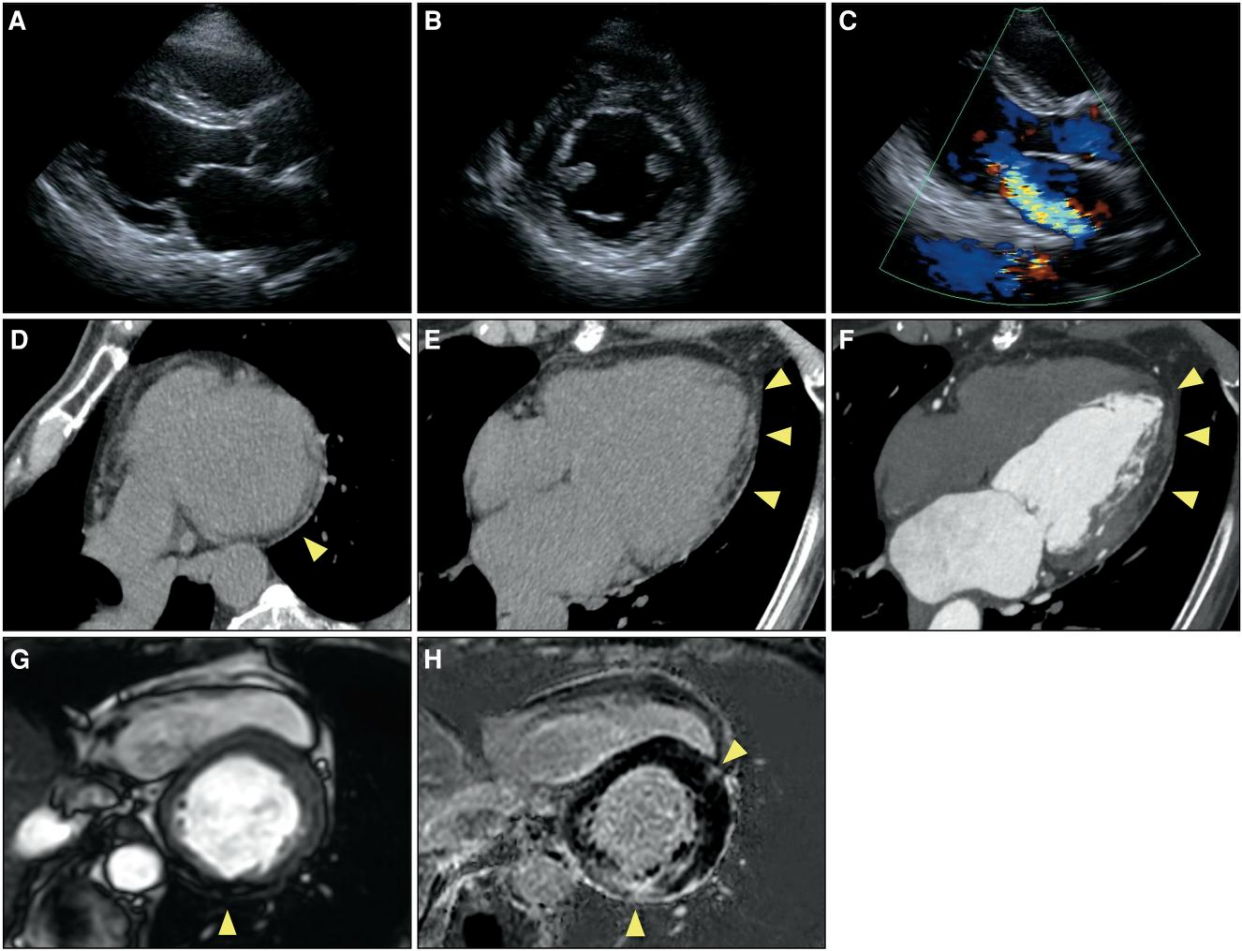
Multimodal imaging findings in 2019. (*A–C*) Transthoracic echocardiography revealing a thinning of the inferior-to-inferolateral wall to 7.3 mm and mild-to-moderate mitral regurgitation. The interventricular septum was 10.7 mm, and the left ventricular hypertrophy was not observed; left ventricular ejection fraction was 60%. (*D* and *E*) Cardiac computed tomography non-contrast images showing low-attenuated areas in the subendocardial-to-epicardial layers of the inferior-to-lateral wall, indicating fatty infiltration (arrowheads). (*F*) Contrast-enhanced images showing poorly enhanced areas, consistent with low-attenuated areas on the non-contrast images (arrowheads). (*G*) On cardiac magnetic resonance imaging, the cine image obtained with the steady-state free precession technique reveals thinning and fat infiltration in the inferior-to-inferolateral wall (arrowhead). (*H*) Transmural late gadolinium enhancement is observed in the thinned inferolateral wall, showing both transmural and mid-myocardial patterns. Additional patchy enhancement is noted in the mid-myocardial region of the anterior junction (arrowheads).

Catheter ablation for paroxysmal atrial fibrillation was performed at a former hospital in 2019. Electrocardiography showed a shortened P_end_Q interval and inverted T waves in leads V3–V6 (*Figure 2B*). Transthoracic echocardiography revealed thinning of the inferior-to-inferolateral wall to 7.3 mm and mild-to-moderate MR. The interventricular septum was 10.7 mm, and LVH was not observed (*Figure 3A–C* and Supplementary material online, *Videos S1*–*S3*). Cardiac computed tomography (CCT) showed no significant coronary stenosis or obstruction but demonstrated extensive fatty infiltration in the subendocardial-to-epicardial layers of the inferior-to-lateral walls (*Figure 3D–F*). On CMR imaging, cine images revealed thinning and fat infiltration in the basal inferior-to-inferolateral wall. Transmural late gadolinium enhancement (LGE) was observed in the thinned inferior-to-inferolateral wall, showing both transmural and mid-myocardial patterns. Additional patchy LGE was noted in the mid-myocardial region of the anterior junction (*Figure 3G* and *H*). T1 mapping analysis was not performed because it was unavailable at that time. ^18^F-fluorodeoxyglucose–positron emission tomography (FDG-PET) showed no active myocardial inflammation. It was difficult to identify the cause of the thinning in the inferolateral wall at that time.

**Figure 4:**
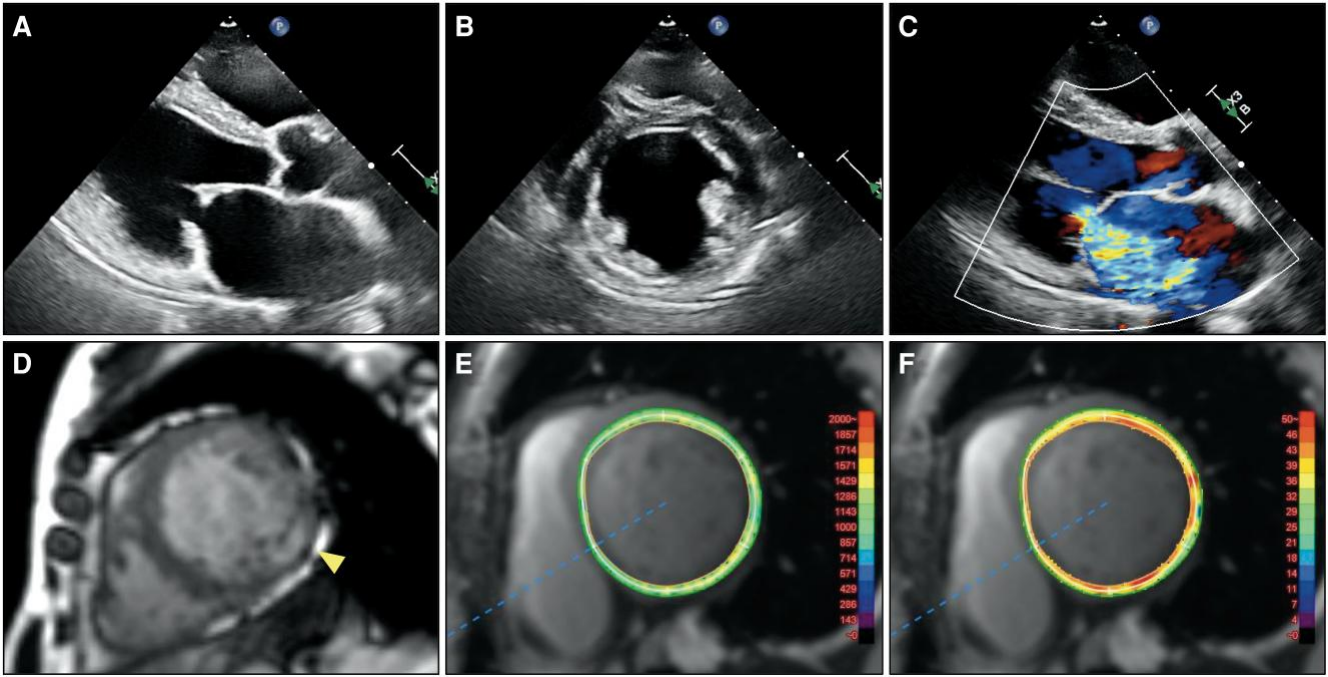
Multimodal imaging findings in 2023. (*A–C*) Transthoracic echocardiography revealing that the left ventricular wall motion was diffusely impaired in addition to the thinned inferior-to-inferolateral wall, and left ventricular ejection fraction was decreased to 42%. Mitral regurgitation became severe. Left ventricular hypertrophy was not identified. (*D*) On cardiac magnetic resonance imaging, transmural late gadolinium enhancement is observed in the thinned inferior-to-inferolateral wall (arrowhead). (*E* and *F*) T1 mapping analysis indicating that native T1 value and extracellular volume are diffusely elevated, including in non-late gadolinium enhancement areas. The mean native T1 value and extracellular volume are 1318 ms (normal: 1182 ± 24 ms) and 40.3% (normal: 23–28%), respectively.

The patient was referred to our hospital for regular follow-up in 2022 and continued to complain of chest pain at rest. Re-examination of coronary angiography revealed no significant coronary stenosis or obstruction. The acetylcholine provocation test was negative for coronary vasospasms. She remained stable for some time; however, she was admitted to our hospital in 2023 with acute decompensated heart failure. On admission, ECG showed intraventricular conduction disturbances and inverted T waves in Leads II, III, aVF, and V4–V6 (*Figure 2C*). Transthoracic echocardiography revealed that LV wall motion was diffusely impaired in addition to a thinning inferior-to-inferolateral wall, and the LV ejection fraction (LVEF) decreased to 42%. Mitral regurgitation became severe owing to mitral annular enlargement and tethering of the posterior mitral leaflet. No LVH was identified (*Figure 4A–C* and Supplementary material online, *Videos S4*–*S6*). Cardiac magnetic resonance imaging revealed transmural LGE in the thinned inferior-to-inferolateral wall. T1 mapping analysis indicated that native T1 value and extracellular volume were diffusely elevated, including in non-LGE areas. Mean native T1 value and extracellular volume were 1318 ms (normal: 1182 ± 24 ms) and 40.3% (normal: 23–28%), respectively (*Figure 4D–F* and Supplementary Method). The FDG-PET revealed the absence of active myocardial inflammation. Cardioprotective medication was initiated, and transcatheter edge-to-edge repair was performed for severe functional MR.

**Figure 5:**
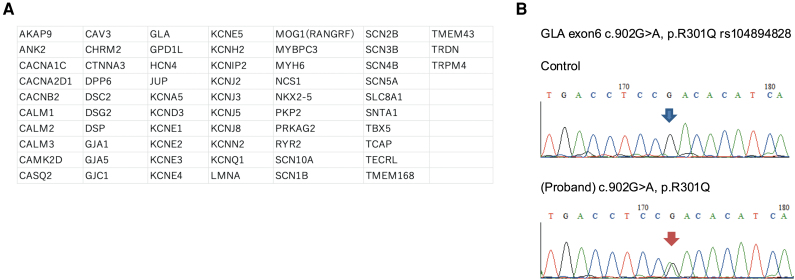
(*A*) Genes included in the customized panel (HPA-14). (*B*) Sanger sequencing confirmed a heterozygous pathogenic *GLA* variant, c.902G>A, p.Arg301Gln.

Despite treatment with transcatheter edge-to-edge repair, the patient was readmitted for heart failure in 2024. Transthoracic echocardiography revealed that MR had improved to a mild level; however, LV function deteriorated, and LVEF decreased to 29%. Electrocardiography monitoring revealed non-sustained ventricular tachycardia. Owing to the family history of dilated cardiomyopathy and fatty infiltration in the LV, arrhythmogenic LV cardiomyopathy was suspected as a differential diagnosis for ongoing LV dysfunction. Subsequently, comprehensive genetic testing using a customized panel identified a heterozygous pathogenic *GLA* variant (c.902G>A, p.Arg301Gln), indicating Fabry disease (*Figure 5A* and *B*).^4^ Further evaluation of Fabry disease revealed anhidrosis and gastrointestinal symptoms. Urinary analysis revealed mulberry bodies, but no proteinuria. Blood tests showed reduced α-galactosidase A activity in white blood cells of 18.3 mmol/mg (normal: 49.8–116.4 mmol/mg) and elevated lyso-Gb3 at 4.65 nmol/L (normal: <0.6 nmol/L), confirming the diagnosis of Fabry disease. Following a multidisciplinary discussion, enzyme replacement therapy (ERT) with recombinant α-galactosidase A was initiated.

## Discussion

We encountered a case of advanced-stage cardiac Fabry disease in which diagnosis was delayed because of the absence of LVH. The patient presented with inferolateral wall thinning and LGE at the time of referral to our hospital, without LVH. Subsequently, LV systolic dysfunction worsened; genetic testing revealed a pathogenic *GLA* variant (c.902G>A, p.Arg301Gln), confirming Fabry disease. This case emphasizes the importance of comprehensive evaluation of cardiac Fabry disease, even without LVH.

In cardiac Fabry disease, LVH primarily results from the lysosomal accumulation of Gb3 within cardiomyocytes owing to α-galactosidase A enzyme deficiency. Direct Gb3 storage increases myocyte volume, leading to cellular hypertrophy. Gb3 accumulation also triggers chronic myocardial inflammation and subsequent fibrosis, impairing diastolic and systolic function.^5^ The absence of LVH makes early identification of cardiac Fabry disease challenging, leading to a delay in diagnosis and the start of ERT or chaperone therapy; these disease-specific treatments have been shown to prevent further deterioration of affected organs and may even reverse existing impairment.^6^ However, as cardiac involvement progresses, the effectiveness of these treatments is limited, worsening prognosis.^7^ Early diagnosis and treatment before irreversible myocardial fibrosis develops is crucial for long-term improvement in cardiac function and prognosis.^8^

Several clinical staging systems for cardiac Fabry disease have been proposed to standardize the assessment of disease severity and guide management. According to a clinical consensus statement by the European Society of Cardiology Working Group on Myocardial and Pericardial Diseases and the European Association of Cardiovascular Imaging, comprehensive multimodality imaging is recommended for diagnosing various cardiac abnormalities, assessing disease severity, and monitoring treatment response in Fabry disease.^2^ In particular, this consensus proposed a classification with four stages: Stage 0 (subclinical damage), Stage 1 (LVH), Stage 2 (myocardial fibrosis), and Stage 3 (heart failure). Del Franco *et al*.^9^ also proposed a classification with four stages. These clinical staging systems emphasized the need for comprehensive evaluation for early diagnosis, allowing early initiation of specific treatments to prevent irreversible fibrosis.

Cardiac Fabry disease can manifest as various ECG abnormalities, even in early stages. Typical findings include a short PQ interval, often <120 ms, or a short P_end_Q interval <40 ms. Other non-specific findings can include QRS prolongation, ST-segment changes, and T-wave abnormalities, which may reflect myocardial injury or conduction system involvement.^10^ In this case, short P_end_Q intervals or other repolarization abnormalities may have provided an earlier clue despite the absence of LVH. Wall thinning in the inferolateral region is a typical TTE finding of cardiac Fabry disease. Global longitudinal strain abnormalities, especially in the inferolateral wall, can indicate subclinical myocardial damage before LVH development or wall thinning^11^; however, this was not measured during the initial workup in this case.

Cardiac magnetic resonance imaging is considered the gold standard for myocardial tissue characterization in patients with cardiac Fabry disease. Late gadolinium enhancement usually shows focal fibrosis, primarily in the inferolateral wall; here, LGE was detected even in the absence of LVH. Low native T1 values are critical findings caused by glycosphingolipid accumulation and often precede LVH and LGE. In the advanced stage, native T1 values become pseudo-normalized or elevated, reflecting increased fibrosis.^12^ In this case, LGE was already present at the time of the first CMR, but T1 mapping analysis was not performed because it was unavailable. The follow-up CMR revealed that native T1 value and extracellular volume were diffusely elevated, including regions without LGE, consistent with diffuse fibrosis.

Extensive fatty infiltration of the LV was observed on CCT and CMR images. Fatty infiltration of the myocardium is a radiologic finding of several types of cardiomyopathies, such as arrhythmogenic cardiomyopathy, dilated cardiomyopathy, and ischaemic cardiomyopathy.^13^ While Gb3 accumulation is the hallmark of Fabry disease and leads to characteristic low native T1 values on CMR, fatty infiltration is not a common finding. However, a recent case report described fatty infiltration of the inferolateral wall in Fabry disease on CCT and CMR, a finding also observed in the right ventricle.^14,15^ This fatty infiltration could be related to fibrofatty replacement in end-stage disease, as seen in ischaemic cardiomyopathy, or as a consequence of Gb3 accumulation.

Advanced-stage cardiac Fabry disease is characterized by severe and often irreversible cardiac injury, leading to refractory heart failure and fatal arrhythmias.^6,9^ Cardiac involvement progresses from LVH to severe LV systolic and diastolic dysfunction. Left ventricular wall motion exhibited diffuse hypokinesis and regional thinning in the inferolateral wall.^3^ This phenotype mimics end-stage hypertrophic cardiomyopathy and dilated cardiomyopathy.^16,17^ However, to our knowledge, there have been no reports of advanced-stage cardiac Fabry disease presenting systolic dysfunction without developing LVH. Even in the advanced stage, it is essential to consider cardiac Fabry disease as a potential diagnosis for LV dysfunction, even in the absence of LVH. In particular, heterozygous female patients tend to present with a late-onset phenotype.^8^ In cases where the family history of Fabry disease is unclear, the disease may progress without being correctly diagnosed, as typical cardiac manifestations such as LVH may not be present. Including *GLA* gene in genetic screening panels for unexplained cardiomyopathies is crucial to prevent diagnostic delays and ensure timely access to disease-specific treatment.

Many pathogenic variants exist for *GLA*; the p.Arg301Gln variant is associated with late-onset or non-classical phenotypes of Fabry disease, primarily affecting the heart and kidneys, with symptom onset in middle age.^18^ Unlike the classic form of Fabry disease, the typical clinical symptom triad (cornea verticillata, neuropathic pain, and angiokeratomas) is uncommon with this variant. Males are more severely affected and present earlier than females. Left ventricular hypertrophy is a common cardiac manifestation in patients carrying this variant, with LV dysfunction without LVH having never been reported.

## Conclusion

Left ventricular hypertrophy is a key feature of cardiac Fabry disease. However, myocardial injury can occur even in the absence of LVH. A comprehensive multimodal imaging evaluation that considers clinical staging is essential for accurate diagnosis of cardiac Fabry disease. It is also crucial to include cardiac Fabry disease in the differential diagnosis of LV dysfunction with inferolateral wall thinning or LGE, even in the absence of LVH, as it can progress to LV dysfunction without LVH in the advanced stages.

## Primary specialities

Paediatrics, nephrology, neurology.

## Supplementary Material

ytag234_Supplementary_Data

## Data Availability

Data sharing is not applicable to this article, as no datasets were generated or analysed in the current study. The data underlying this article are available in the article and online Supplementary material.

## Lead author biography

Masataka Suzuki is a cardiologist at Kobe City Nishi-Kobe Medical Center, Kobe, Japan. His main clinical and research interests include diagnostic cardiac imaging.
