# Supplementary material for: A diagnostic challenge of advanced-stage cardiac Fabry disease without left ventricular hypertrophy: a case report
Source: Eur Heart J Case Rep. 2026 Mar 20;10(4):ytag234. doi: 10.1093/ehjcr/ytag234 (PMC13098152; doi:10.1093/ehjcr/ytag234)
Supplement: ytag234_Supplementary_Data [file ytag234_Supplementary_Data.zip › Supplementary method 20260122.docx]

**Supplementary method**

Cardiac magnetic resonance (CMR) imaging was performed on a 3T scanner (MAGNETOM Vida, Siemens Healthcare, Erlangen, Germany) with an 18-channel coil.

Late gadolinium enhancement (LGE) imaging was performed 10–15 minutes after the intravenous injection of a gadolinium-based contrast agent (meglumine gadoterate; 0.1 mmol/kg) at the rate of 4 mL/s, followed by a 20–30 mL saline flush. A phase-sensitive inversion recovery (PSIR) turbo fast low-angle shot (turbo-FLASH) sequence was utilized. Typical parameters were: repetition time (TR)/echo time (TE) 809.6/1.28 msec, flip angle 55°, thickness 7 mm, field of view (FOV) 360 × 340 mm², matrix 156 × 224. The optimal inversion time (TI) was determined using a TI scout (Look-Locker) sequence to null the signal of normal myocardium.

T1 mapping imaging was performed with a modified Look-Locker inversion-recovery (MOLLI) sequence that used true fast imaging with steady-state precession (TrueFISP) with a fixed sampling scheme (5(3)3) to measure native T1 of the myocardium and acquired mid short-axis images of the left ventricle. Typical parameters were: TR/TE 261.66/1.12 msec, minimum TI 100 msec, TI increment 80 msec, FOV 360 × 247 mm, matrix case dependent, flip angle 35°; phase partial Fourier 7/8, thickness 8 mm, number of acquisitions 3.
